# An Integration of RNA Sequencing and Network Pharmacology Approaches Predicts the Molecular Mechanisms of the Huo-Xue-Shen Formula in the Treatment of Liver Fibrosis

**DOI:** 10.3390/ph18020227

**Published:** 2025-02-07

**Authors:** Keying Jiang, Jianfeng Bao, Zhonghan Lou, Fei Liu, Keyang Xu, Hiu Yee Kwan

**Affiliations:** 1 Centre for Cancer and Inflammation Research, School of Chinese Medicine, Hong Kong Baptist University, Hong Kong, China; 23482826@life.hkbu.edu.hk; 2 Hangzhou Xixi Hospital, Zhejiang Chinese Medical University, Hangzhou 310020, China; zjbjf1972@aliyun.com (J.B.); lzh123807@outlook.com (Z.L.); liufeitingyue@163.com (F.L.); 3 State Key Laboratory of Quality Research in Chinese Medicine, Faculty of Chinese Medicine, Macau University of Science and Technology, Macau 999078, China; 4 Institute of Systems Medicine and Health Sciences, School of Chinese Medicine, Hong Kong Baptist University, Hong Kong, China; 5 Institute of Research and Continuing Education, Hong Kong Baptist University, Shenzhen 518000, China

**Keywords:** Huo-xue-shen, liver fibrosis, machine learning, molecular docking, network pharmacology

## Abstract

**Background**: Liver fibrosis is a prevalent, chronic inflammatory condition characterized by the excessive accumulation of extracellular matrix components and, primarily, collagen in the liver. Huo-xue-shen (HXS) has proven effective for the treatment of liver fibrosis. However, the mechanism is yet to be deciphered. **Methods**: Network pharmacology, machine learning algorithms and RNA-seq were used to predict the immune-treated targets and mechanisms associated with HXS in liver fibrosis. Molecular docking was employed to screen for effective agents based on the drug–compound–hub gene network in HXS, aiming to identify the most critical bioactive compound in HXS for the treatment of liver fibrosis. **Results**: A total of 100 immune-treated targets (ITTs) of HXS were found to significantly regulate the PI3K-Akt signaling pathway and the MAPK signaling pathway. Among these, CDKN1A, NR1I3, and TUBB1, which can concurrently interact with quercetin, were associated with the prognosis of liver fibrosis, indicating that HXS may inhibit or reverse HSC activation primarily by suppressing neutrophil extracellular trap formation, stimulating oxidative phosphorylation and promoting thyroid hormone synthesis in the regulation of the liver microenvironment. **Conclusions**: Our study suggests that HXS may delay the progression of liver fibrosis by targeting multiple pathways, as shown by the network pharmacology and transcriptome profiling used to examine the liver immune environment. Quercetin, its key ingredient, likely plays an important role by mediating the CDKN1A, NR1I3, and TUBB1 signaling pathways. Overall, our findings provide a new perspective on the potential biological mechanisms of this traditional Chinese medicine formula.

## 1. Introduction

Liver fibrosis is a wound-healing response triggered by chronic liver injury, characterized by the accumulation of excessive extracellular matrix (ECM) and the formation of fibrotic scars [1]. Chronic liver disease (CLD) is recognized as a global health burden, affecting about 1.5 billion individuals and resulting in around 2 million deaths every year [2].

Non-alcoholic fatty liver disease (NAFLD) and hepatitis B virus infection (HBV) are two major causes of CLD [3]. NAFLD is attributed to the accumulation of fat in the liver, which can lead to progressive fibrosis and hepatocellular carcinoma (HCC). HBV plays a crucial role in liver fibrosis and cirrhosis [4]. Conventional approaches to managing liver fibrosis include weight loss, reduction of alcohol consumption, antiviral therapy and other lifestyle changes, all of which can effectively slow the progression of the condition. Combined with dietary control and exercise [5], Resmetirom, an oral thyroid hormone receptor-β (THR-β) agonist, which was approved for the treatment of advanced liver fibrosis (stage-F2 to -F3 fibrosis) with noncirrhotic NASH in the USA, has shown promising results in improving fat metabolism in the liver. Studies show that it also significantly recovers liver function compared to other treatments [6]. However, its long-term effects and safety profiles are still under evaluation, leaving the potential side effects and overall effectiveness uncertain.

Traditional Chinese medicine has been used for thousands of years, providing abundant empirical evidence and clinical experience related to various diseases, including liver fibrosis [7]. Natural products with antifibrotic potential and minimal adverse effects are among the leading strategies for the prevention and reversal of liver fibrosis. The exploration of drugs and natural extracts for therapeutic purposes has garnered significant interest in recent decades [7]. Numerous studies have confirmed that many Chinese herbal extracts are effective for managing liver fibrosis. Huo-xue-shen (HXS), a particular traditional Chinese formula, is a good example. This formula was developed and refined based on a previous study demonstrating that huo-xue-shen-shi-fang (HXSSF) significantly prevents the progression of liver fibrosis in rats [8]. It consists of *Poria cocos (Schw.)* Wolf (Chinese name: Fulin), *Bupleurum chinensis* DC (Chaihu), *Atractylodes macrocephala* Koidz (Baizhu), *Coix lacryma-jobi* L. (Yiyiren), *Astragalus membranaceus* (Fisch) Bge (Huangqi), *Carthamus tinctorius* L. (Shuihonghuazi), *Codonopsis pilosula* (Franch) Nannf (Dangshen), *Angelica sinensis* (Oliv.) Diels (Danggui), *Amomum villosum* (Zingiberaceae) (Sharen), *Curcuma zedoaria* (Christm) Roscoe (Ezhu), *Paeonia lactiflora* Pall (Baishao), *Glycyrrhiza uralensis* Fisch (Gancao), *Trionyx sinensis* Wiegm (Biejia), *Citrus reticulata* Blanco (Zhike), *Paeonia lactiflora* Pall (Shuifeiji), *Dioscorea* thumb (Dilong), *Gynostemma pentaphyllum* (Thunb.), *Makino* (Linxiaohua), *Cyperus rotundus* L. (Xiangfu), *Curcuma aromatica* Salisb (Yujin), *Platycodon grandifloras* (Jacq.) A. DC. (Jiegeng), *Lablab purpureus* (L.) Sweet (Baibiandou), *Artemisia capillaris* Thunb. (Yinchen), *Hedyotis diffusa* Willd. (Chuipencao), and *Scutellaria baicalensis* Georgi (Liushenqu). This Chinese medicinal composition addresses the therapeutic need to alleviate the disease patterns of asthenia of healthy qi and sthenia of pathogenic factors in the general treatment of liver fibrosis [8]. Dangshen, huangqi, fulin, and baizhu work together to replenish qi, while zhike promotes qi movement to soothe the middle. Baishao nourishes yin and softens the liver, and biejia, along with the earth dragon, helps to free collateral vessels and activate the blood. Shuihonghuazi, lingxiaohua, and ezhu target blood stasis, whereas yinchen and chuipencao act as dampness-draining agents with anti-icteric properties. Danggui is included for its blood-tonifying effects. The combination of xiangfu and yujin is recognized for soothing the liver and regulating qi. Jiegeng aids in lung diffusion and qi regulation, while chaihu alleviates liver depression. Liushenqu resolves stasis, and yiyiren, sharen, and baibiandou strengthen the spleen and drain dampness. Gancao harmonizes the actions of the entire prescription. On the whole, this prescription employs both elimination and reinforcement, addressing the supplementation of deficiency, in line with the theory of treating both the liver and spleen from *Huangdi Neijing*.

Increasing evidence suggests that the active herbal compounds of HXS, such as paeoniflorinand [9] and saikosaponin [10], exhibit a variety of pharmacological effects, inhibiting the action of hepatic stellate cells and reducing the deposition of extracellular matrix protein. However, it is important to highlight that studies on the HXS formula have primarily focused on individual drugs in isolation, lacking comprehensive descriptions of the potential mechanisms behind its antifibrotic effects. Additionally, it is complex to use a conventional approach to identify the key protein targets of the HXS formula, which comprises twenty-four drugs that may interact with multiple proteins and signaling pathways.

Network pharmacology and machine learning algorithms are advanced approaches in drug discovery. They are valuable tools for identifying active drugs that target multiple biological pathways, potentially improving outcomes. More importantly, network pharmacology employs a “one gene, one target, and one disease” approach that enables us to better understand the biological mechanism of the synergistic drug action. It is a cost-effective strategy that involves systematically analyzing genes, proteins, pathways, and responses to evaluate the effects of the formula treatment, allowing for the integration and interpretation of data through suitable algorithms. In this study, we employed network pharmacology, machine learning, and molecular docking to reveal the potential antifibrosis targets of the HXS formula and to identify the key bioactive compound responsible for its mechanism of action.

## 2. Results

### 2.1. Revealing the Temporal Immune Cell Landscape in Liver Fibrosis

**Figure 1:**
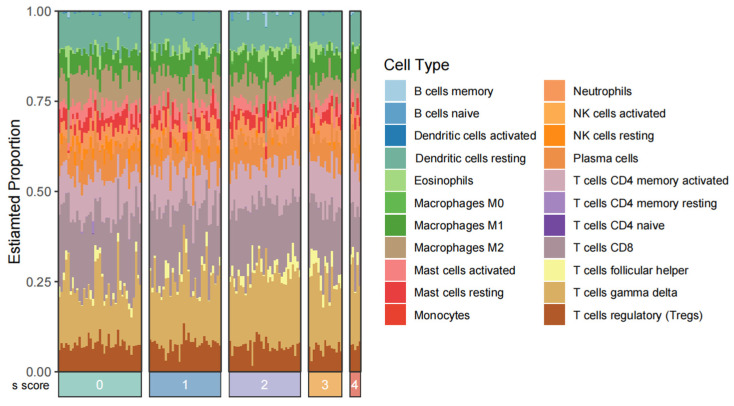
Investigation of immunomodulation in the process of liver fibrosis. Landscape of immune cells between liver tissues in 5 different stages in GSE84044, estimated by Cibersortx.

As the immune response changes during the progression of liver fibrosis, we analyzed the proportions of 22 immune cell types across 124 samples from GSE84044 using the Cibersortx algorithm with LM22 signature files. According to the Scheuer scoring system, liver fibrosis was categorized into five stages, with S0–S1 indicating no significant liver fibrosis. Our findings reveal that T cell gamma-delta and CD8 T cells were predominant components of the liver microenvironment across all stages (Figure 1). Additionally, the proportions of follicular helper T cells, T cell gamma-delta, resting NK cells, and macrophages (including M0, M1, and M2) exhibited significant differences among the five stages (Table 1). The results from the Kruskal–Wallis test indicated that the immune environment evolves throughout the progression of liver fibrosis.

### 2.2. Constructing the Weighted Gene Co-Expression Modules Network

**Figure 2:**
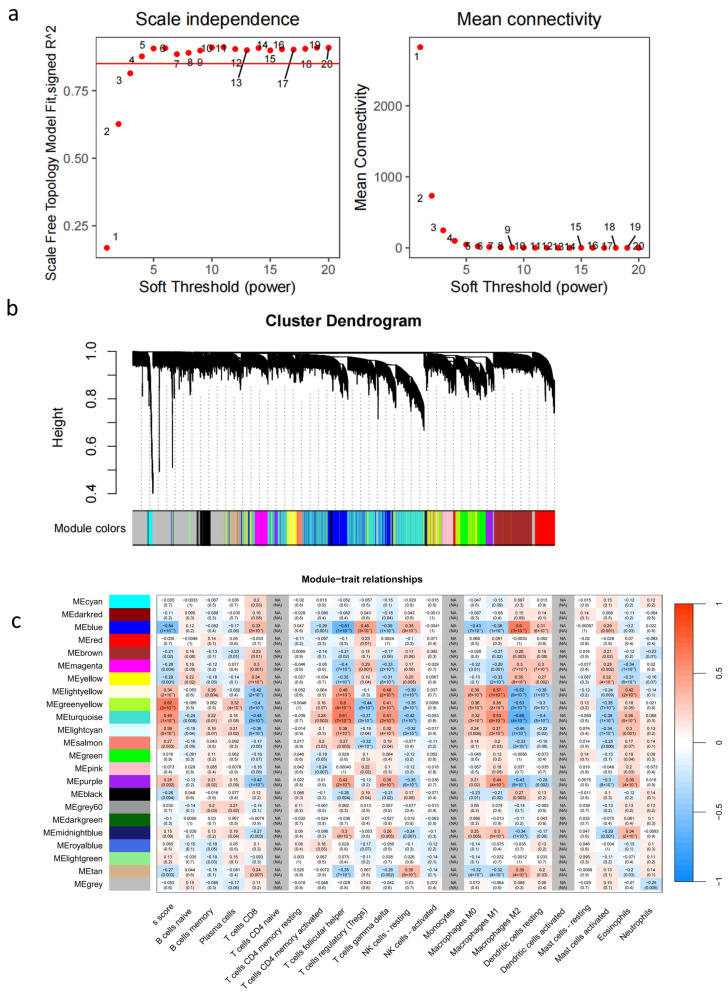
Weighted gene co-expression network analysis: (**a**) determination of the optimal soft threshold utilized for gene clustering; (**b**) dendrogram of all differentially expressed genes from the dynamic tree cut; (**c**) analysis of the relationships between modules and traits, involving Scheuer score “S” and the infiltration of the 22 immune cell types.

Next, we constructed a hierarchical clustering tree using the gene expression data, converted into the TPM format, for 17,335 genes from 124 samples. A power value (β)  =  5 was chosen in this study to align with the scale-free topology criterion, keeping the co-expression modules network scale-free (Figure 2a). The scale-free topology fits index reached values above 0.9 for the soft-thresholding power. The mean connectivity decreased as the soft threshold increased. This trend indicates that higher soft threshold values lead to a more selective connection pattern among nodes, contributing to the emergence of a scale-free topology (Figure 2b). The combination of the two scatter plots suggests that the network retains its scale-free characteristics by fostering a few highly connected nodes while reducing the overall connection density. The genes were then divided into 21 co-expression modules according to the dynamic tree cut (Figure 2c). However, among the 17,335 genes, 3280 genes did not fit within a distinct group and were categorized into the grey module and excluded in the following study.

### 2.3. Revealing the Module-Traits Correlation in Liver Fibrosis and Immune Cell Infiltration

Then, we further evaluated the relationship between the 20 gene modules and fibrosis Scheuer ‘S’ scores, and immune cell infiltration. Twelve modules show fibrotic-association evidence with *p* < 0.05, but only three modules, MEblue, MEgreenyellow and MEturquoise among them have strong correlation (|Cor| > 0.5) with immune cell (T cell follicular helper (Tfh) cells and M2 macrophages) simultaneously, containing 5742 genes in total. They are recognized as immune related targets against liver fibrosis (Figure 2c).

### 2.4. Identifying the Potential Targets of HXS

**Figure 3:**
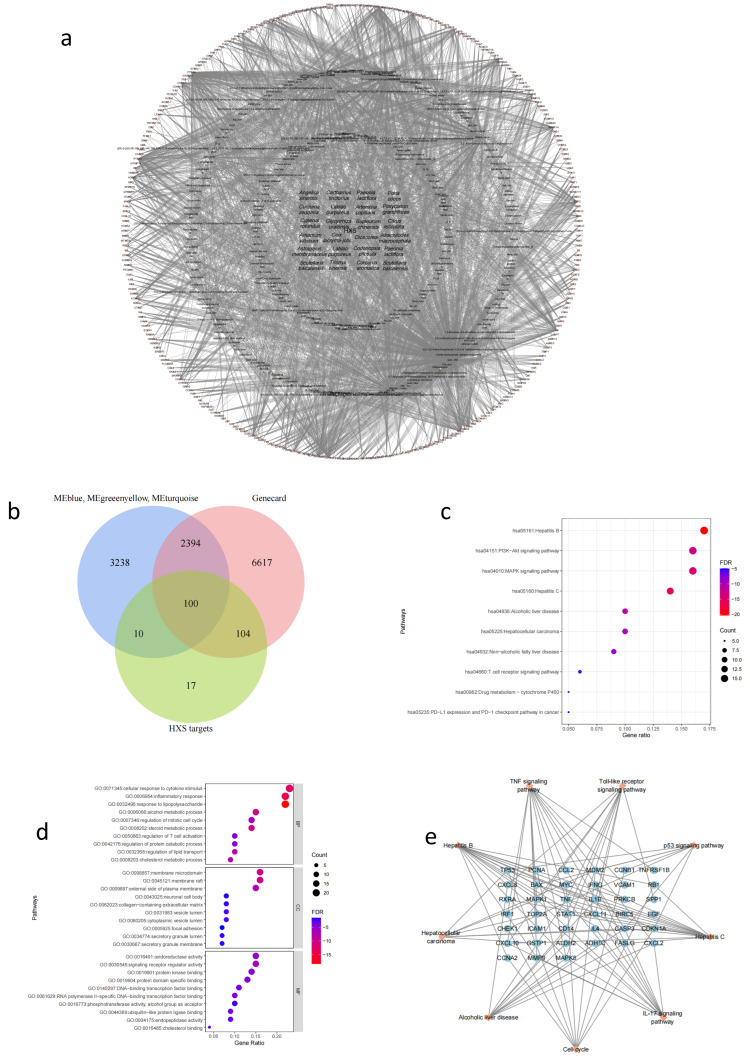
Multidimensional network graph analysis and enrichment analysis of the network pharmacology targets. (**a**) Disease–herb–active component–target network diagram. The yellow diamond-shaped nodes represent the main active components of HXS, grey square node represent the compositions of HXS and the red nodes represent the potential targets treating liver fibrosis. (**b**) A Venn diagram of the targets both in HXS, WGCNA, and liver fibrosis targets recorded in the GeneCards database. The overlapping parts indicate the immune-treated targets (ITTs) according to which HXS treats liver fibrosis. (**c**) Top 10 KEGG pathways of ITTs. (**d**) Top 10 GO terms of ITTs. (**e**) Pathway–targets network. The orange diamond-shaped nodes represent KEGG pathways, and the blue nodes represent genes enriched in those signaling pathways.

We used network pharmacology to search for possible targets of action. A total of 206 active ingredients and 291 potential targets were retrieved (Figure 3a). Next, we explored a total of 9215 targets for liver fibrosis (LF) using the Genecards database [11], to identify the targets of HXS in suppressing liver fibrosis. We then employed WGCNA analysis to extract the immune-related targets associated with HXS and the microenvironment. A favorable micro-immune environment can facilitate the reversal of fibrosis. The WGCNA analysis focuses on identifying the gene clusters related to the progression of liver fibrosis and immune cells. Network pharmacology was used to screen the targets of the formula for disease-related genes from the database to identify the targets of HXS that influence the immune environment. We constructed the immune-HXS-LF target set, which includes a total of 100 common genes, identified as the immune-treated targets (ITTs) of HXS therapy (Figure 3b).

### 2.5. A Functional Enrichment Analysis of ITTs

We carried out a Gene Ontology annotation and enrichment analysis with Metascape (available online: https://metascape.org/gp/index.html (accessed on 29 January 2025)). The top GO functional analysis and KEGG pathways in the ITTs were identified. Generally, the GO annotation revealed that HXS affects a broad range of biological processes, cellular components, and molecular functions regulated by immune cells. Ten GO terms were independently listed in these three categories, all with low *p*-values and significant target enrichment. The results indicate that ITTs are strongly associated with processes involving immune cells, such as cellular response to cytokine stimulus, inflammatory response, and response to lipopolysaccharide (Figure 3d). Based on the KEGG database, ten significantly enriched pathways were displayed in the bubble map. The size and color of the nodes in the bubble graph correspond to the number of related genes and their *p*-values. The colors range from red to blue, indicating *p*-values from high to low, while the sizes of the nodes reflect the gene ratio associated with each pathway. The results of the KEGG pathway enrichment analysis suggest that multiple pathways and mechanisms are involved in the action of HXS against liver fibrosis, including the hepatitis B pathway, PI3K-AKT signaling pathway, and MAPK signaling pathway (Figure 3c). These signaling pathways involve inflammation and ECM deposition. Cumulatively, these pathways are closely related to the therapeutic effects of HXS against liver fibrosis. The targets–pathways network is helpful for understanding their interactions (Figure 3e). This illustrates the multifaceted nature of HXS’s action in regulating the immune response, as it may influence several signaling cascades that contribute to hepatocellular carcinoma and alcoholic liver disease.

### 2.6. Screening of ITTs in the Verification Cohort by Protein–Protein Interaction Network Construction

**Figure 4:**
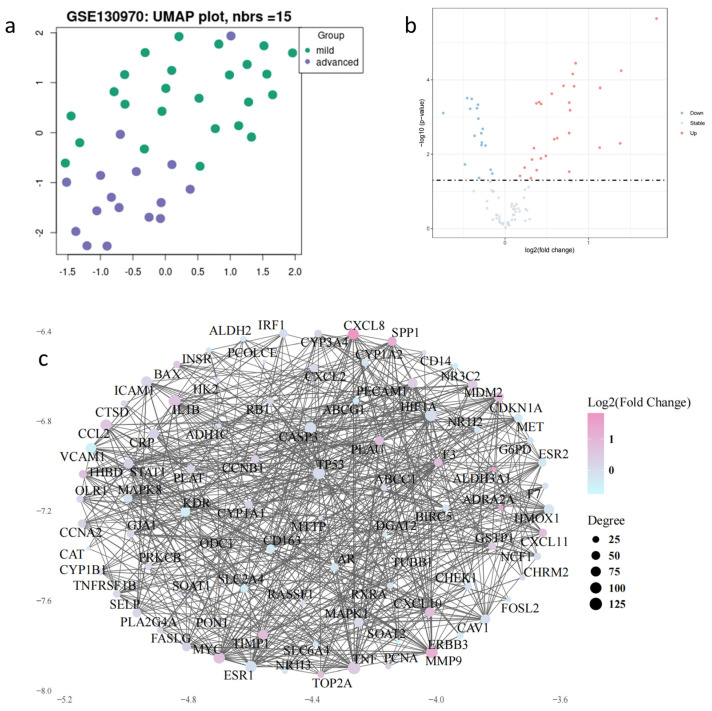
Differential genes expression analysis: (**a**) distribution of mild and advanced liver fibrosis samples in GSE130970 in UMAP. (**b**) Volcano plot of 44 differential genes in ITTs. (**c**) Protein–protein interaction network constructed from 88 ITTs that can be screened in GSE13090.

Next, we retrieved the human gene dataset GSE130970 from the GEO database as a verification cohort for liver fibrosis. The 24 liver tissues graded 0 in the fibrosis stage were defined as the “mild group”, and 16 fibrotic samples graded 3 or 4 were defined as the “advanced group”. The global structure of these 40 samples was tested using the UMAP approach. The UMAP plot shows that the two groups are heterogeneous. A total of 88 genes from 100 common genes in ITTs can be found in this dataset. Of these, 27 genes are upregulated and 17 are downregulated significantly (Figure 4b). In addition, we also explored the protein-to-protein interactions of the 88 genes using the STRING database (Figure 4c). The PPI network provides a comprehensive visualization of the protein interactions, revealing how expression changes correlate with protein connectivity. The use of color shading based on log FC values allows for the identification of key upregulated proteins, while node sizes based on degree values highlight potential hub proteins critical to the biological processes under study. This multifaceted representation aids in understanding complex biological interactions and can guide further functional analyses of the identified proteins.

### 2.7. Predicting the Potential Hub Genes and Related Clinical Values of the Binding of ITTs by Machine Learning Algorithms

**Figure 5:**
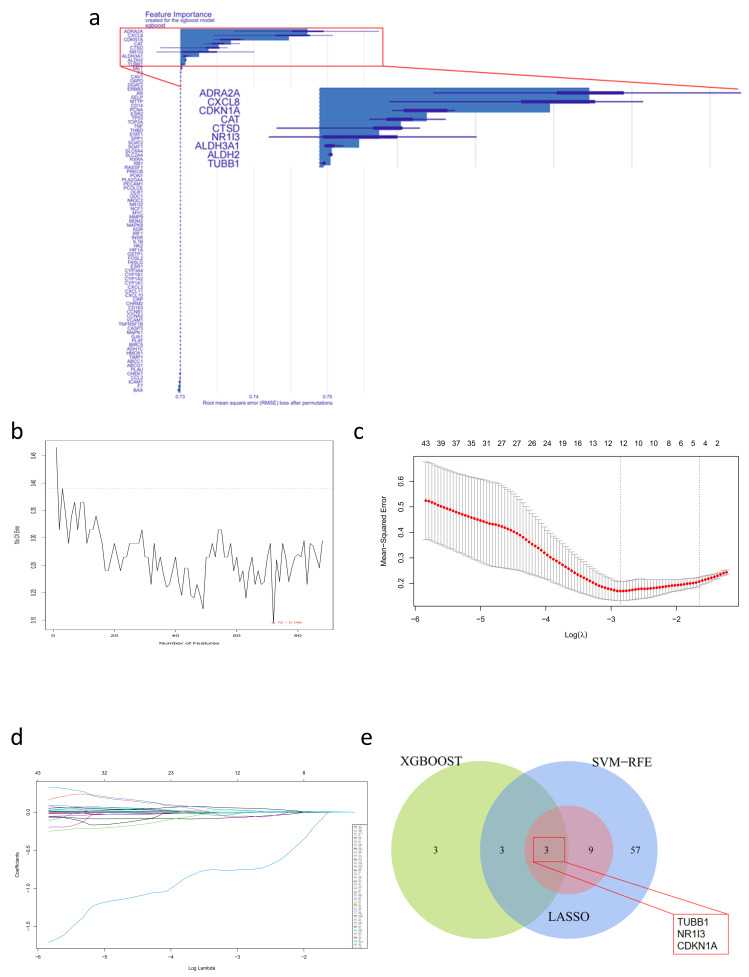
Feature selection to find hub genes in ITTs. (**a**) Features’ importance ranking from the XGBoost analysis, with a longer bar representing the variables that have more influence on the outcome variables. (**b**) Measurement of the performance of the cross-validated SVM-RFE technique. When there were 72 variables, the model had the minimum error. (**c**) Cross-validation to select the optimal tuning parameter log (Lambda) in the LASSO regression analysis. The left dotted line represents the minimum error of the model, and the right represents the minimum number of features within the range of error tolerance. In this study, we chose the model with the minimum error. (**d**) LASSO coefficient profiles of ITTs. The LASSO was applied for the regression of high dimensional predictors. The method utilizes an L1 penalty to shrink some unnecessary regression coefficients to zero. (**e**) The Venn diagram of targets shared by the XGBoost, SVM-RFE, and LASSO algorithms.

We employed three machine learning algorithms to identify core inflammatory-related therapeutic and diagnostic features based on ITTs through comprehensive attribute selection. We aimed to confirm that the hub genes are critical for the efficacy of HXS in treating liver fibrosis. A total of 9, 72, and 12 ITTs were selected using the XGBoost, SVM-RFE, and Lasso algorithms independently (Figure 5a–d; Table 2). The bar chart ranks the importance of features based on their contribution to the outcome in the XGBoost model, where longer bars indicate higher importance. In the XGBoost analysis, a total of nine ITTs were identified as having high importance, as indicated by the relatively long bars (Figure 5a).

The selection of the top 72 features by the SVM-RFE method was based on achieving the minimum cross-validation error and high accuracy. This decision is grounded in principles of model performance, complexity management, and the ability to generalize to unseen data. Figure 5b effectively illustrates the trade-offs involved in feature selection and emphasizes the importance of cross-validation in determining the optimal configuration.

The cross-validation results for LASSO regression are shown, plotting error against the logarithm of lambda. The left dotted line indicates the minimum error, while the right dotted line marks the minimum number of features that fall within an acceptable error range. By selecting the model with the minimum error, the study aims to balance model accuracy and complexity. The chosen lambda is critical, as it influences which features are retained in the final model (Figure 5c). The LASSO coefficient profiles of candidate ITTs illustrate the coefficients of different candidate ITTs obtained through LASSO regression (Figure 5d). Each line represents the coefficient of a variable as a function of the tuning parameter (lambda). The LASSO method applies an L1 penalty, effectively shrinking some coefficients to zero and thus selecting a subset of important predictors. Twelve variables with coefficients that remain non-zero as lambda increases are deemed important for the model, while those that shrink to zero are considered less relevant.

The Venn diagram illustrates the overlap of hub genes identified by the LASSO, SVM-RFE, and XGBoost algorithms. The three shared genes among the methods strengthen the validity of these candidates, as they are consistently identified across the following different analytical approaches: CDKN1A, NR1I3, and TUBB1 (Figure 5e). This figure collectively demonstrates a rigorous approach to feature selection for modeling. By integrating multiple methods, the study ensures that the selected ITTs are both biologically relevant and statistically significant, thus enhancing the reliability of the clinical prognosis models in predicting outcomes.

**Figure 6:**
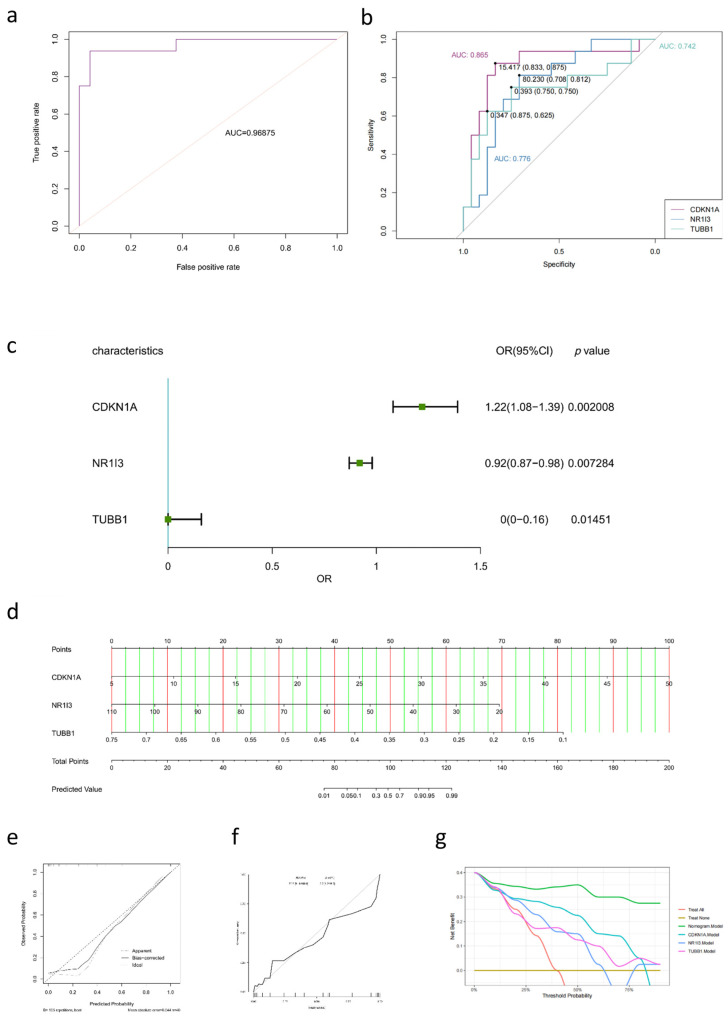
Development of Nomogram for estimating the probability of advanced liver fibrosis and its predictive performance in verification cohort. (**a**) ROC curve of nomogram. (**b**) AUCs comparison among the hub genes independently. (**c**) Forest plot of the univariate logistic regression analysis of the hub genes in liver fibrosis. (**d**) The nomogram incorporated the related TPM expression of the hub genes in established for assessing the probability of advanced liver fibrosis. (**e**) Calibration curves of the occurrence of advanced liver fibrosis in the nomogram. The *x*-axis and *y*-axis indicate the predicted probability and actual probability respectively. The dotted line indicates the best predicted value. (**f**) ROC curve of constructed nomogram yielded an AUC of 0.969 with Brier score of 0.062. (**g**) The DCA curves of the four prediction models, nomogram and the three hub genes, respectively. The net benefit curves for these prognostic models are presented. *x*-axis suggests the threshold probability for advanced liver fibrosis and y-axis suggests the net benefit.

In addition, the three hub genes are theorized to have pertinent classification values in advanced liver fibrosis. Therefore, an ROC curve analysis was performed on the verification cohort to evaluate the diagnostic accuracy of the model, and it was found that the area under the curve (AUC) reached 0.96875 (Figure 6a), indicating that the classifier using the three hub genes together was highly reliable. While the classification ability was weaker when addressing each gene separately (Figure 6b), it remains well-classified, with CDKN1A achieving an AUC of 0.865, NR1I3 an AUC of 0.776, and TUBB1 an AUC of 0.742.

Next, the differences between advanced fibrosis (fibrosis stages 3–4) and mild fibrosis (fibrosis stage 0) were analyzed, categorized by the three hub genes. As shown in Figure 6c, the univariate logistic regression analysis indicates that the three hub genes affect the occurrence of liver fibrosis, as follows: CDKN1A with an odds ratio (OR) of 1.22 (95% CI: 1.08–1.39), NR1I3 with an OR of 0.92 (95% CI: 0.87–0.89), and TUBB1 with an OR of 0.95 (95% CI: 0.80–1.16).

Taken together, a predictive nomogram combining the three hub genes for advanced fibrosis in the verification cohort was constructed. A summed score is calculated as the totaled scores of the hub genes, which corresponds to the probability of advanced fibrosis on the basal axis (Figure 6d). For example, in a patient whose CDKN1A score is 15, NR1I3 count is 55, and TUBB1 score is 0.4, the total points scored would be 116.5, resulting in an advanced fibrosis probability of approximately 97%.

The calibration plot for the probability of advanced fibrosis showed optimal agreement between the predictions made by the nomogram and the actual observations. The calibration curve of the model was relatively close to the ideal curve, demonstrating good consistency between the predictions based on the hub genes together and the actual observations (Figure 6e).

Meanwhile, the calibration of the nomogram for predicting the fibrosis probability was also analyzed. The area under the receiver operating characteristic (ROC) curve (AUC) for the constructed nomogram was 0.969, with a Brier score of 0.626, as shown in Figure 6f. This indicates a good correlation between the observed and predicted liver fibrosis.

Additionally, a decision curve analysis (DCA) was performed to further evaluate the clinical value of the nomograms in predicting advanced liver fibrosis (Figure 6g). This analysis quantifies the net benefit probability within a threshold range of 0 to 87.5%. The lines for each gene and the nomogram model demonstrate that clinical benefits can be achieved by using these indices. Patients will experience a higher benefit when the net benefit is more significant under the same threshold probability.

In simpler terms, DCA exhibits a direct correlation between the decision curve and the net benefit of the models’ clinical decisions, which is independent of other factors. Clinical decisions are more favorable when there is a greater distance from the “Treat none” line. In the verification cohort, DCA revealed that the nomogram was markedly superior in predicting the risk of advanced fibrosis, suggesting that the nomogram model is an effective assessment tool.

### 2.8. Analysis of the Molecular Mechanism of the Hub Gene in HXS Treatment Using RNA-Seq

**Figure 7:**
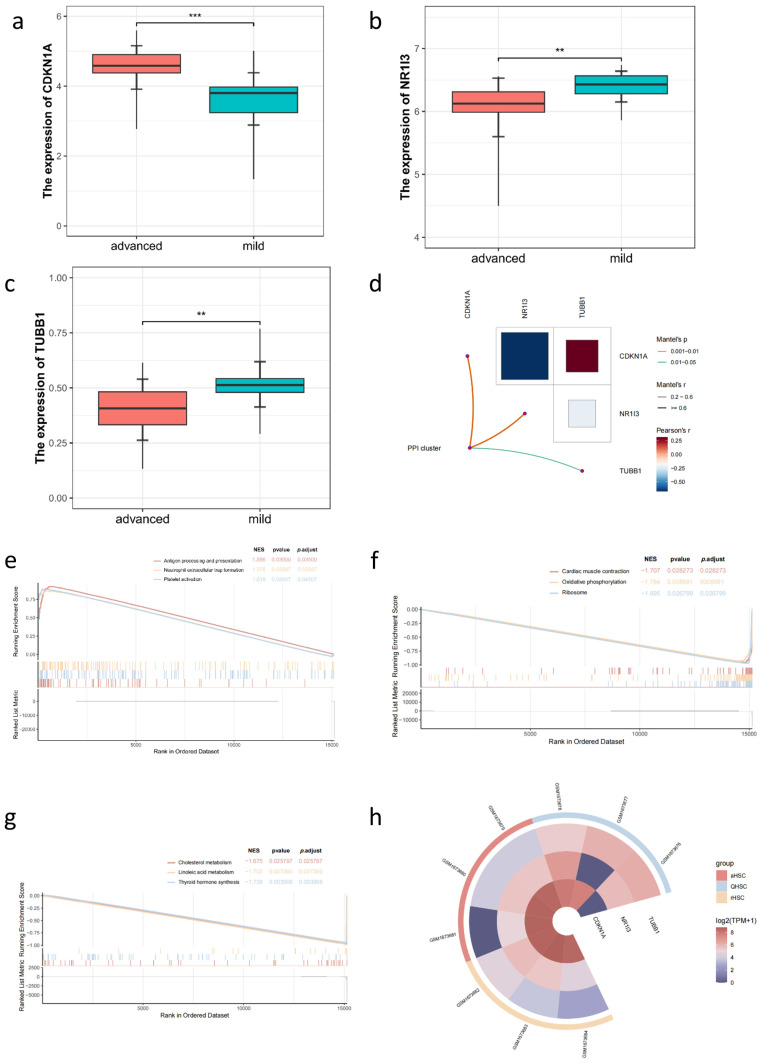
The expression of the three hub genes in liver fibrosis. (**a**–**c**) The expression profiles of the three hub genes in the verification cohort (*n* = 40). (**d**) The relationship between the ITTs and hub genes in the advanced liver fibrosis samples. (**e**–**g**) Curves of the GSEA enrichment highlighting the enriched pathways of the (**e**) upregulated CDKN1A and downregulated (**f**) NR1I3 and (**g**) TUBB1. (**h**) Heatmap displaying the relative expression levels of hub genes among quiescent HSC (qHSC), activated HSC (aHSC), and reverted HSC (rHSC). **: *p* < 0.01; ***: *p* < 0.001.

The expression levels of the three hub genes, CDKN1A, NR1I3, and TUBB1, in immune cell infiltration types (ITTs) were subsequently analyzed in the verification cohort (Figure 7a–c). The results indicate a significant difference in the expression of these hub genes (*p* < 0.05) between the advanced and mild fibrosis groups. Specifically, CDKN1A is upregulated in the advanced patient group, while NR1I3 and TUBB1 are downregulated. This observation is consistent with the pathology of advanced liver fibrosis, where CDKN1A, a cell cycle regulator, may be involved in promoting cellular responses to stress or damage, potentially contributing to fibrosis progression. Conversely, the downregulation of NR1I3 and TUBB1 may indicate a loss of functions that typically regulate cellular proliferation and structural integrity, suggesting a shift in the cellular environment associated with advanced fibrosis. Additionally, the expression levels of these hub genes correlate with the ITTs, as illustrated by Mantel’s test. The correlation analysis shows the relationships among the ITTs cluster and the three hub genes, with orange and green lines connecting significant pairs to highlight strong relationships between variables. For instance, the line connecting “ITTs” and “CDKN1A”, as well as the line between “ITTs” and “NR1I3”, emphasizes their tight relevance. In contrast, the relationship between ITTs and TUBB1 is weaker (*p* < 0.05).

The Pearson correlation coefficients (r) displayed within the matrix quantify the strength and direction of the linear relationships between variable pairs. For example, a correlation of 0.25 between “CDKN1A” and “TUBB1” suggests a weak positive relationship, indicating that as one variable increases, the other tends to increase as well. Conversely, a correlation of −0.63 between “CDKN1A” and “NR1I3” indicates a strong negative relationship, suggesting that increases in “CDKN1A” are associated with decreases in “NR1I3” (Figure 7d). In Figure 7d, additional information can be gleaned from the correlation heatmap network. The color gradients represent pairwise comparisons of the hub genes, with red squares symbolizing positive correlations and blue squares symbolizing negative correlations. This visualization allows for a quick assessment of the relationships among the hub genes and their interactions with ITTs. The color and width of the lines linking the ITTs and the hub genes represent both the statistical significance and the Mantel’s r statistic independently, providing a comprehensive view of the strength and significance of these relationships. Overall, Figure 7d not only highlights the correlations among the hub genes but also emphasizes the varying degrees of significance and strength of the relationships in the context of liver fibrosis, aiding in the understanding of the underlying biological mechanisms.

GSEA was employed to identify the biological pathways and processes associated with liver fibrosis. By analyzing gene expression profiles from liver tissues, we aimed to uncover pathways that are significantly altered in fibrotic conditions. This approach enables us to understand the underlying biological mechanisms contributing to liver fibrosis, which is crucial for developing targeted therapies. The expression trends of individual genes within these pathways correlate with the overall enrichment scores observed. For instance, CDKN1A, which is involved in “antigen processing and presentation”, “platelet activation”, and “neutrophil extracellular trap formation”, exhibited increased expression in fibrotic liver tissues (Figure 7e), aligning with the observed enrichment scores. This suggests that the activation of these pathways may play a significant role in the pathophysiology of liver fibrosis. Conversely, the low expression of NR1I3 is associated with pathways such as “cardiac muscle contraction”, “oxidative phosphorylation”, and “ribosome” (Figure 7e). Meanwhile, the suppressed expression of TUBB1 is enriched in pathways related to “cholesterol metabolism”, “thyroid hormone synthesis”, and “linoleic acid metabolism” (Figure 7f). These findings highlight the diverse biological processes involved in liver fibrosis and underscore the importance of these pathways in understanding the disease’s progression and potential therapeutic targets. Collectively, these findings could have potential implications for hepatic stellate cell (HSC) activation in the progression of liver fibrosis (Figure 7g). To further verify the changes in expression levels of the three hub genes, we utilized another published RNA-seq dataset, GSE68001, which focuses on primary HSCs from humans. The data reveal that NR1I3 and TUBB1 are downregulated when HSCs transition from a quiescent state to an activated state, while CDKN1A is upregulated during this process. In contrast, there is minimal change in the expression levels of these genes when HSCs revert from an activated state back to quiescence (Figure 7h). These observations suggest that the activation of HSCs is closely linked to the expression dynamics of these hub genes, further supporting their potential roles in the pathophysiology of liver fibrosis.

### 2.9. Construction of the Pharmacal Network, Molecular Docking, and Molecular Dynamics

**Figure 8:**
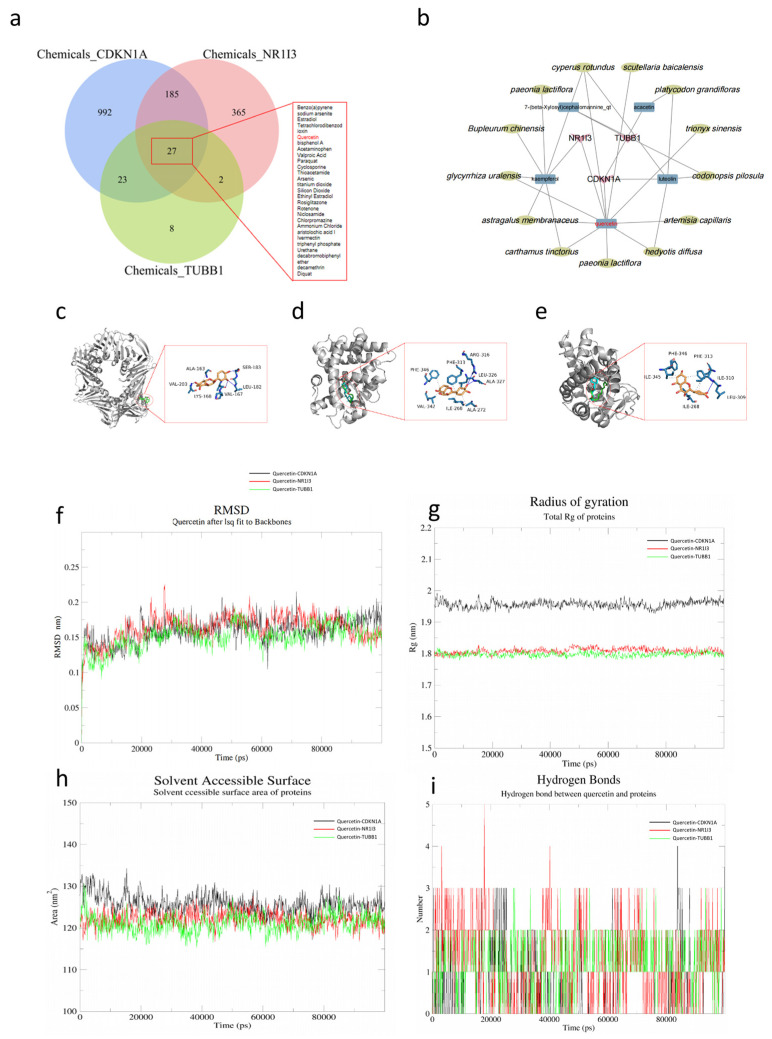
Exploration of the interaction between hub genes as therapeutic targets and quercetin. Twenty-nine drugs were obtained from the CTD database that were associated with the hub genes. (**a**) The Venn diagram of interacting chemicals shared by CDKN1A, NR1I3, and TUBB1 retrieved from the CTD database. (**b**) The interaction network of the three hub genes, related agents, and drugs. (**c**–**e**) Molecular docking results of quercetin with (**c**) CDKN1A, (**d**) NR1I3, and (**e**) TUBB1, with the hydrogen bonding interactions shown in blue line. (**f**) RMSD plot of the hub proteins in complex with quercetin at an 100 ns time interval. (**g**) The Rg plot of the hub proteins in complex with quercetin at an 100 ns time interval. (**h**) Solvent-accessible surface area (SASA) values of the three complexes. (**i**) Number of hydrogen bonds in the three complexes.

To identify the most critical bioactive compound in HXS for treating liver fibrosis by targeting CDKN1A, NR1I3, and TUBB1, we studied gene–drug interactions based on CTD (Comparative Toxicogenomics Database, available online: https://ctdbase.org/ (accessed on 29 January 2025)). In total, 27 chemicals were found to interact with the hub genes (Figure 8a). The herbal–compound–hub gene network in HXS also specifies the key ingredients targeting these hub genes (Figure 8b). The herb–compound–hub gene network analysis of HXS provided important insight into the relationships between herbal ingredients and their potential targets in the context of liver fibrosis. Among the various active compounds identified, quercetin is predicted as the common matching ligands of the hub genes and emerges as the most prominent ingredient in this network (degree = 11). This mutual information found in the public database and the high degree indicate that quercetin interacts with the hub genes, suggesting its significant role in influencing biological pathways relevant to liver fibrosis. Furthermore, its interactions with these targets underscore quercetin’s potential to modulate fibrotic processes and promote liver health.

Therefore, to validate the interaction affinity of quercetin with the proteins that the three hub genes encode, which can also be considered as the hub proteins in this study, molecular docking was conducted. Based on the docking scores and protein–small molecule interactions, quercetin binds at the active pockets of the proteins. Quercetin binds to CDKN1A (PDB ID: 1AXC) with a binding energy of −7.3 kcal/mol (Figure 8c), with no co-crystalized ligand found. Microstegiol was utilized as a benchmark to provide a comparable scoring reference, as has been suggested as exhibiting the most favorable binding interactions with the CDKN1A protein [12], with a higher binding energy of only −5.9 kcal/mol under the circumstances of this study (Figure S1a). Further, NR1I3 (PDB ID: 1XV9) and TUBB1 (PDB ID: 1XVP) share the same co-crystallized ligand, pentadecanoic acid. The binding energy between NR1I3 and quercetin is −8.5 kcal/mol (Figure 8d), which is lower than that of NR1I3 with its co-crystallized ligands (−7 kcal/mol) at the same binding site (Figure S1b). As TUBB1 binds to quercetin, the binding energy between them is −7.8 kcal/mol (Figure 8e), which is also lower than the binding energy observed with its co-crystallized ligands at that binding site, measured as −6.9 kcal/mol (Figure S1c).

Based on the molecular docking results, a 100 ns molecular dynamics simulation was conducted on the interactions of the quercetin–hub protein complex. Root means square deviation (RMSD), radius of gyration (Rg), Solvent Accessible Surface Area (SASA) and number of hydrogen bonds are evaluated to explore their stability and dynamics of quercetin upon the proteins binding. The RMSD of the hub proteins stabilize after 30ns between 0.07 and 0.32 nm, showing stable RMSD values and good protein stability (Figure 8f). The Rg plots were also performed to explore the compactness of the quercetin–hub proteins complexes. Rg provides information of the root mean square distance of atoms from the center of mass, offer deep insights into the protein stability overall. The Rg plots for the quercetin–hub proteins are shown in Figure 8g. The average Rg score of the complex with CDKN1A is 1.95nm, while the score of that with NR1I3 or TUBB1 is 1.8. In addition, SASA analysis illustrates that quercetin–CDKN1A is stable after fluctuation for 10ns, with average SASA score 125 nm^2^, while the SASA score of the other two stabilizes in around 122 nm^2^. As for the change in the number of the hydrogen bonds, the quercetin–NR1I3 ranges the most during 100 ns (1~5). This number of the other two ranges from 1 to 2.

## 3. Discussion

Liver fibrosis hinders the daily activities of patients, reducing their quality of work and social life. It is often diagnosed late, when treatment may be less effective due to asymptomatic progression. However, few strategies approved for reversion directly target fibrosis itself. The activation of hepatic stellate cells is crucial in the development of fibrosis and involves various immune mediators, but the precise mechanisms remain poorly described. Additionally, the extracellular matrix components and signaling molecules in the liver microenvironment influence the behavior of the immune system and fibrogenesis. Studying the interactions among these factors is important for drug development.

The utilization of traditional Chinese medicine is common in preventing liver fibrosis because of its safer therapeutic efficacy and fewer side effects. It is believed to support liver detoxification, restore liver function, and mitigate the phenotype of fibrosis by using herbs, such as milk thistle (Silybum marianum), bupleurum, and chrysanthemum. Additionally, it emphasizes a balance between Yin and Yang, which may be beneficial for regulating immune cell behavior in the liver and, thus, reducing chronic inflammation.

In this study, three hub genes, CDKN1A, NR1I3, and TUBB1, were identified from 88 ITTs using three machine learning algorithms. CDKN1A, which encodes p21, is a cyclin-dependent kinase inhibitor. Studies have shown that inhibiting p21 expression has the potential to control the progression of liver fibrosis and promote tissue regeneration by eliminating senescent cells [13,14,15]. Another study found that CDKN1A-induced hepatocytes produce CXCL14, which accumulates macrophages and may contribute to the acceleration of the fibrotic process while inhibiting the clearance of activated HSCs [16]. Interestingly, our single-gene GSEA results suggest that upregulated CDKN1A is primarily enriched in the formation of neutrophil extracellular traps [17], a pathway that impairs endothelial restoration by upregulating the gene and, consequently, suppressing the cell cycle. This indicates that CDKN1A is a promising target for restoring endothelial function in the regression of liver fibrosis [18] and should be considered when developing new drugs to treat the disease in the future.

NR1I3, which encodes the constitutive androstane receptor (CAR), is a nuclear receptor involved in the regulation of drug metabolism, including encoding cytochrome P450 enzymes [18]. It is primarily expressed in the liver and plays an important role in regulating hepatocyte proliferation in collaboration with the STAT3 signaling pathway [19]. Studies have shown that the overexpression of CAR in HepaRG-CAR cells is associated with an increased expression of genes involved in oxidative phosphorylation and mitochondrial energy metabolism [20]. Increasing oxidative phosphorylation have several implications for liver fibrosis. Enhanced oxidative phosphorylation may improve mitochondrial function and energy production, which could promote hepatocyte survival and regeneration. However, excessive oxidative stress resulting from increased oxidative phosphorylation can also lead to cellular damage and inflammation, potentially exacerbating fibrotic processes. In our GSEA single-gene enrichment results, the genes associated with the oxidative phosphorylation pathway are downregulated alongside the lower expression of NR1I3 under advanced liver fibrosis conditions. This suggests that a decline in CAR expression may contribute to impaired mitochondrial function and energy metabolism in the liver, further complicating the progression of fibrosis.

Previous studies have highlighted that the inhibition of oxidative phosphorylation (OXPHOS) significantly causes hepatocyte damage, immune cell activation, and inflammation, indicating that impaired oxidative phosphorylation can lead to increased cellular stress and inflammation, which are critical factors in the development of liver fibrosis [21]. Additionally, impaired induction of NR1I3 (CAR) is associated with liver failure following excessive tissue loss as well [22]. The death or damage of hepatocytes may trigger a wound-healing response, which leads to the activation of hepatic stellate cells and the excessive production of ECM proteins [20], ultimately resulting in the formation of fibrotic scars and the progression of liver fibrosis. Although there is no direct evidence supporting the relationship between the suppression of NR1I3 and advanced liver fibrosis, NR1I3 remains a promising target for the treatment of liver fibrosis.

TUBB1 encodes β1-tubulin, a protein that is particularly important for the formation of the platelet cytoskeleton and the maintenance of platelet shape. The gene has been identified as a potential therapeutic target for patients with hepatocellular carcinoma (HCC) with high EME (epigenetic and epitranscriptomic module eigengene) scores [23]. Based on our current study, significant reduction in TUBB1 expression inhibits thyroid hormone synthesis [24], linoleic acid metabolism, and cholesterol metabolism. Research has demonstrated that abnormal thyroid hormone levels are associated with various liver diseases by regulating lipid homeostasis in the liver. Adipokines such as TNF-α, leptin, and resistin can be disrupted by lower levels of thyroid hormone in circulation, exacerbating liver fibrosis [25,26]. Moreover, excessive accumulation of linoleic acid in the liver can increase oxidative stress, which is widely recognized as a key driver of liver fibrosis [27]. Limited studies have shown a direct link between linoleic acid metabolism and liver fibrosis; however, an inverse association has been noted between dietary linoleic acid intake and the risk of significant liver fibrosis [28]. Additionally, linoleic acid metabolites such as HYA have been found to ameliorate liver fibrosis in both cross-sectional and preclinical studies [29]. Similarly, disturbances in cholesterol homeostasis have the potential to increase accumulation of free cholesterol in the liver, consequently leading to the development and progression of liver fibrosis in non-alcoholic fatty liver. Similarly, disturbances in cholesterol homeostasis can lead to an accumulation of free cholesterol in the liver, consequently promoting the development and progression of liver fibrosis in non-alcoholic fatty liver disease [30]. Despite these findings, research on the function of TUBB1 remains limited, particularly regarding its potential mitigating role in reversing liver fibrosis.

In our pursuit to explore potential therapeutic agents of the drugs in HXS interacting with these hub genes, we have identified quercetin as a promising candidate. The results of molecular docking and dynamics simulation indicate a strong affinity between quercetin and the target proteins. Quercetin is one of the most active representatives of flavonoid compounds in traditional Chinese medicine, and numerous studies have confirmed its pharmacological activity against drug-induced liver injury, NAFLD, liver fibrosis, liver cancer, and other liver diseases [31]. However, the expression of CDKN1A is generally found to be enhanced by quercetin during normal liver regeneration [32]. Its effects under conditions of liver fibrosis may differ from those observed in normal regeneration, necessitating further research for clarification. Additionally, quercetin intake has been shown to upregulate the expression of NR1I3 [33], a gene that plays a role in regulating the signaling targets involved in drug metabolism and lipid metabolism. This regulation contributes to managing lipid levels and, subsequently, slowing the progression of liver fibrosis. We provide a new perspective for TUBB1 as a potential therapeutic target of quercetin in liver fibrosis.

The present study has certain limitations that require further investigation. While this study offers statistically convincing results, HXS formula’s ability to fully encapsulate the experiential essence of traditional Chinese medicine in treating liver fibrosis requires further validation through a more extensive collection of medical records demonstrating its clinical effectiveness. In addition, most patients with liver fibrosis would lose their access to timely treatment because of the lack of sensitive diagnostic methods, which would limit the treatment efficacy of this formula. On the other hand, the hub genes and pivotal signaling pathways are identified through network pharmacology and bioinformatics approaches, their biological functions, both in vivo *and* in vitro, require further elucidation. Additionally, while quercetin is identified through online resources, pharmacological experiments are necessary for more robust preclinical observations. Our investigation applied the CIBERSORTx algorithm to retrieve immune cell infiltration data. However, it should be noted that other methods, such as single-cell RNA sequencing (scRNA-seq), could also be used to verify these findings. scRNA-seq can provide a more reliable immune landscape in the liver microenvironment, allowing for the identification of cell subsets and a deeper understanding of their diverse functions. More importantly, case control and cohort study need to be performed to ensure its actual therapeutic effect in practice [34].

## 4. Materials and Methods

### 4.1. Patients and Samples

The liver biopsy gene expression profiles of GSE84044, GSE130970 and primary human quiescent HSCs gene expression profile of Data series were obtained from the GSE68001, with GSE84044 representing a microarray dataset produced using the Affymetrix Human Genome U133 Plus 2.0 Array (Thermo Fisher Scientific, Waltham, MA, USA). It includes 124 samples from chronic hepatitis B (CHB) with pathological Scheuer Score ‘S’ 0–4. Using the annotation documents from the respective platforms, we annotated the gene expression profiles in each dataset. This process yielded a matrix with sample names as rows and gene symbols as columns, ready for subsequent analysis. Simultaneously, we extracted the clinical characteristics of patients with CHB to enrich our dataset. To gain detailed prediction into the relation between the drugs target protein and the prognosis of patients with liver fibrosis, high throughput bulk RNA sequencing of samples with fibrosis stage 0 (*n* = 24), 3 and 4 (*n* = 16) in GSE130970 dataset was used in constructing following prognostic models. Illumina HiSeq 2500 (Homo sapiens) (Illumina, Inc., San Diego, CA, USA) was employed for sequencing the samples. This approach indicates valuable insights for identifying hub genes of HXS and unveiling underlying mechanism that could pave the path for the drug development of treating liver fibrosis forward. To gain the specific change in gene expression in HSCs, the dataset of GSE68001 contains the transcriptomic profile of primary human quiescent HSC and in vitro activated HSC (*n* = 9) was utilized. Its raw data analysis processed by GPL13667 platform of Affymetrix Human Genome U219 Array (Thermo Fisher Scientific, Waltham, MA, USA).

### 4.2. Analysis of Immune Cell Infiltration

The sequencing data of GSE84044 were downloaded through R package GEOquery (version 2.72.0) and then normalized in TPM format by R package gprofile2 (version 0.2.3) and DGEobj.utils (version 1.0.6), which were used to determine the abundance of 22 subtypes of immune cell. These fractions represent the cell composition of liver immune microenvironment in different states. Kruskal–Wallis test was then applied to analyze their differences between various states.

### 4.3. Weighted Gene Co-Expression Network Analysis (WGCNA) and Module Detection

WGCNA is commonly used to identify key genes in gene networks, co-expressed modules correlated with clinical phenotype. The GSE84044 dataset was used to obtain modules related to the Scheuer score “S” and immune cell highly. In addition, the WGCNA package was utilized to construct a co-expression network for the 17,334 genes. Then, the scale-free topology of the network was evaluated for multiple values of the β shrinkage parameter. β = 5 was used on scale-free topology criterion. Genes with similar expressed patterns were clustered into a module applying the “dynamic tree cutting” algorithm when setting the minimum size of 20. Simultaneously, the correlation analysis between module genes and sample traits, Scheuer score “S”, and immune cell infiltration was conducted using Pearson’s correlation test.

### 4.4. Screening of Active Compounds and Related Targets of HXS

The active compounds of HXS were retrieved using the Traditional Chinese Medicine Systems Pharmacology Database (TCMSP, available online: https://old.tcmsp-e.com/tcmsp.php (accessed on 29 January 2025)) and a Bioinformatics Annotation Database for Molecular Mechanism of Traditional Chinese Medicine (BATMAN-TCM, available online: http://bionet.ncpsb.org.cn/batman-tcm/#/home (accessed on 29 January 2025)). Active compounds were filtered based on criteria such as an oral bioavailability (OB) of at least 30% and a drug-likeness (DL) of no less than 0.18. The corresponding targets for these active compounds were collected from the “Related Targets” section. For compounds without target information, SuperPred (available online: https://prediction.charite.de/ (accessed on 29 January 2025)) was further used to predict their targets.

### 4.5. Construction of the Herbal–Compound–Target Network

Cytoscape, a network visualization tool, was utilized to construct a network of herbal active compounds and their corresponding targets. The potential active compounds of HXS and their respective targets were entered into Cytoscape version 3.8.0. Within this network, each node symbolizes a drug, component, or target, and the connecting lines represent their interactions.

### 4.6. Determination of Liver Fibrosis Prediction Targets

Information about the disease targets associated with liver fibrosis came from the GeneCards database (available online: https://www.genecards.org/ (accessed on 29 January 2025)). Keyword “Liver fibrosis” was used to search for liver-fibrosis-related targets from the database.

### 4.7. GO Enrichment and KEGG Pathway Analyses

To investigate the biological functions of ITTs in the antifibrotic effects of HXS, Gene Ontology (GO) analysis and Kyoto Encyclopedia of Genes and Genomes (KEGG) pathway data were obtained using the Metascape database (available online: https://metascape.org/gp/index.html (accessed on 29 January 2025)). A GO analysis was utilized to forecast biological processes (BPs), cellular components (CCs), and molecular functions (MFs), whereas the KEGG pathway analysis pinpointed crucial signaling pathways. The GO and KEGG data were subsequently imported into R (version 4.3.1) for visualization and further analysis.

### 4.8. Data Collection and Processing

The transcriptome profiling data for hepatic samples were downloaded from dataset GSE130970 in the Gene Expression Omnibus (GEO) database based on GPL16791. The RNA-seq data in the TPM format was downloaded from the Supplemental File. This dataset contains 24 samples in fibrosis stage 0 and 16 samples in fibrosis 3 or 4 in total, which are classified in mild group and advanced group respectively. Quantile normalization of gene expression was done by “normalizabetweenarrays” fuction of “limma” package (version 3.62.2), and log2 conversion was done subsequently. Then, the “limma” package was used to investigate the log2Foldchange value for ITTs with *p*-values. The relative hub gene expression of the mild and advanced tissues was visualized by “ggplot2” package (version 3.5.1).

### 4.9. Construction of the Protein–Protein Interaction (PPI) Network

ITTs were uploaded into the STRING database (available online: https://cn.string-db.org/ (accessed on 29 January 2025)) for PPI analysis. The analysis was conducted by selecting “Homo sapiens” organism and a medium interaction score of 0.400. The results of the PPI were utilized to construct a protein–protein interaction network. The default edge table of the PPI results was also exported from Cytoscape 3.8.0, with the excess nodes removed.

### 4.10. Construction of ITT Diagnostic Model

The XGBoost classifier (version 2.1.3), support vector machine-recursive feature elimination (SVM-RFE), and least absolute shrinkage and selection operator (LASSO) regression were utilized to identify hub genes associated with liver fibrosis prognosis. XGBoost efficiently addresses binary classification tasks by providing direct insight into feature importance. The parameter selection for hub genes in this analysis adhered to the default settings, as outlined in the XGBoost documentation (available online: https://xgboost.readthedocs.io/en/stable/R-package/xgboostPresentation.html (accessed on 29 January 2025)). Significant features were identified, resulting in an increase in accuracy greater than 0.73. SVM-RFE is a robust machine learning algorithm that distinguishes positive and negative instances by eliminating less informative features derived from the SVM model. The “e1071” (version 1.7−16), “glmnet” (version 4.1−8), and “caret” (version 6.0−94), packages were employed to construct the SVM-RFE model, optimizing variable selection based on the minimum 10-fold cross-validation (CV) error. LASSO regression was implemented using the “glmnet” package to reduce data dimensionality while retaining valuable prognostic features. The optimal lambda value (0.005628), determined through ten-fold cross-validation, was used to build the model, which explained 99% of the deviance, indicating its effectiveness in predicting outcomes and selecting relevant features. The intersection of the results from XGBoost, SVM-RFE, and LASSO were used to identify the valid hub genes for this research.

### 4.11. ROC, Logistic Regression, and Nomogram Model Construction

To assess the fibrosis severity of patients with liver disease, the hub genes were merged into a nomogram. A nomogram model was built to predict the state of liver fibrosis using the “rms” package. It was constructed based on discrimination with calibration curves, which describe the assessment of the predictive ability. Decision curve analysis is useful when it is applied to make better clinical decisions. The curves were plotted to describe the net benefit based on the nomogram, CDKN1A, NR1I3, and TUBB1. The net benefit determines whether clinical decisions made based on a model will bring more benefits than harm. Univariable logistic regression analysis was performed using “advanced fibrosis vs. mild fibrosis” as binary outcomes in separate models. The odds ratio (OR), its 95% confidence interval, and their related statistical significance for each hub gene were calculated and examined with the generalized linear model (glm) in the R program. A forest plot was then constructed to display the odds ratios and the 95% CI for the hub genes in the “advanced fibrosis vs. mild fibrosis” groups.

### 4.12. Biological Characteristics Identification of Hub Genes

In order to identify the statistical significance of differences between two groups of data, the Wilcoxon rank sum test was used to evaluate the differences between the mild and advanced groups. All *p*-values were examined on a two-sample basis, and a *p*-value less than 0.05 was proposed as statistically significant. To assess the relationships between the ITTs’ interactions and the hub genes in the advanced state, the Mantel test was performed with the use of the Vegan package. The relationships were assessed with Pearson’s correlation. To study the potential function of the hub genes, a Gene Set Enrichment Analysis (GSEA) function of the “clusterProfiler” package (version 4.14.3) was used. Statistical significance is defined as *p* < 0.05 for the enrichment visualization. A heatmap of the normalized expression of the HSCs in the three different states (qHSC, aHSC, and rHSC) was generated with the “pheatmap” package (version 1.0.12) of R.

### 4.13. Molecular Docking

Molecular docking is widely applied in drug discovery to investigate receptor–ligand recognition processes. It theoretically integrates modeling and affinity to examine molecular interactions and binding modes. AutoDock 4 (version 1.5.6) was utilized to process the molecular docking of quercetin with the hub proteins, forecasting the interaction patterns and strength of the binding between them. The following procedure was carried out: (a) Quercetin was acquired from the Protein Data Bank (PDB, available online: http://www.rcsb.org/ (accessed on 29 January 2025)) in the mol2 format and then used in Chembio3D software (version 12.0) for energy minimization. AutoDockTools (version 1.5.6) was employed to incorporate hydrogen atoms, compute charges, and assign charge values. The ligand’s flexibility was configured using the Ligand option, and the file was subsequently saved in the “pdbqt” format. (b) The proteins NR1I3 and TUBB1 and their affiliated co-crystalized ligands, pentadecanoic acid, were acquired from the PDB. The structural information for the CDKN1A protein was also acquired from this database without an affiliated co-crystalized ligand being found. These proteins and their related ligands were imported into PyMOL (version 2.3.0) software to extract the original ligands and remove water molecules. Subsequently, the proteins were imported into AutoDockTools (version 1.5.6) to add hydrogens, calculate charges, and assign charges. The atom types were set as Macromolecule and saved as “pdbqt” format files. (c) In AutoDockTools (version 1.5.6), the grid box was positioned on the original ligand of the protein. (d) The docking box size was set to 30 × 30 × 30 for the quercetin–NR1I3 and quercetin–TUBB1 complexes, the same for pentadecanoic acid–NR1I3 and pentadecanoic acid–TUBB1, and to 20 × 20 × 20 for the quercetin–CDKN1A complex. Other parameters maintained their default values. (e) The results are presented using PyMOL software.

### 4.14. Molecular Dynamics Simulations

We utilized Gromacs v2022.03 software to simulate the molecular dynamics of the complex obtained through molecular docking for 100 ns, employing the CHARMM36 force field. The specific steps and parameter settings were as follows: (a) The complex in the “pdb” format was converted to the “gro” format, serving as the initial structure for the MD simulation. (b) Using AmberTools22 software (version 22.08), the Generalized Amber Force Field (GAFF) was applied to the small molecule. The molecule was hydrogenated, and the RESP potential was calculated with Gaussian 16 W. The potential data were then incorporated into the molecular dynamics system topology file. (c) A three-point transferable intermolecular potential (TIP3P) solvent was selected to solvate the complex. Protein atoms were ensured to be at least 1.2 nm from the edge of the water box, and the system charge was neutralized by adding appropriate amounts of Na+ and Cl−. (d) The steepest descent algorithm was employed for energy minimization (EM) to stabilize the system. (e) Solutes were confined in an isothermal isochoric (NVT) ensemble, gradually heating the system from 0 K to 300 K, followed by equilibration at 300 K and 1 bar in an isothermal isobaric (NPT) ensemble. (f) A temporal molecular dynamics simulation of the complex was performed for 100 ns, with the simulated trajectories saved for subsequent analysis. Based on the MD simulation results, we calculated the root mean square deviation (RMSD), radius of gyration (Rg), solvent-accessible surface area (SASA), and hydrogen bonding (H-bonds) of the complex. The RMSD and Rg values were used to calculate the Gibbs free energy using the built-in “g_sham” and “xpm2txt.py” scripts of Gromacs v2022.03.

### 4.15. Statistical Analysis

We used the chi-square test for the qualitative data and the Student’s *t*-test for the quantitative data between the two groups. To analyze the differences among the five groups, we utilized one-way ANOVA. We adjusted the *p*-values using the Benjamini–Hochberg (BH) method. For the correlation analysis, we employed Pearson’s linear correlation. We considered a *p*-value of less than 0.05 to be statistically significant.

## 5. Conclusions

In summary, we demonstrate that the HXS formula could improve liver function and decrease the activation of hepatic stellate cells (HSCs), exerting an antifibrotic effect based on network pharmacology and RNA-seq analyses. The potential molecular mechanisms involve the hepatitis B, PI3K-Akt, and MAPK signaling pathways through the inhibition of 100 identified target interactions (ITTs). Overall, our results suggest that HXS could alleviate liver fibrosis through multiple targets and related pathways, with the three hub genes—CDKN1A, NR1I3, and TUBB1—serving as biomarkers.

## Figures and Tables

**Table 1:** The results of Kruskal–Wallis test between liver tissues in 5 different states in GSE84044. NA—not applicable.

Cell Type	Q-Value
B Cells—Naive	0.440668844
B Cells Memory	0.120343655
Plasma Cells	0.440668844
T Cells CD8	0.128790774
T Cells CD4 Naive	NA
T Cells CD4 Memory—Resting	0.209861339
T Cells CD4 Memory—Activated	0.431720624
T Cells Follicular Helper	0.000727023
T Cells Regulatory (Tregs)	0.128790774
T Cells Gamma Delta	0.005773282
NK Cells—Resting	0.000727023
NK Cells—Activated	0.306767723
Monocytes	NA
Macrophages M0	0.012278784
Macrophages M1	0.000727023
Macrophages M2	0.0003552
Dendritic Cells—Resting	0.209861339
Dendritic Cells—Activated	NA
Mast Cells—Resting	0.856945295
Mast Cells -Activated	0.306767723
Eosinophils	0.08105074
Neutrophils	0.306767723

**Table 2:** Selected ITTs from the XGBoost, SVM-RFE, and Lasso algorithms independently.

Model Construction	Feature Selection
XGBoost	ADRA2A, CXCL8, NR1I3, CDKN1A, CAT, CTSD, ALDH3A1, ALDH2, TUBB1
SVM-RFE	BAX, CDKN1A, CXCL8, TIMP1, PCNA, ADRA2A, ALDH2, ESR2, MMP9, CCNA2, PLA2G4A, NR1I2, PLAU, CCNB1, CCL2, FASLG, TNF, ERBB3, CHEK1, NCF1, PECAM1, IL1B, NR3C2, STAT1, GSTP1, KDR, MET, F3, CXCL11, PLAT, PCOLCE, SOAT1, NR1I3, CXCL10, G6PD, CD14, CRP, SOAT2, DGAT2, RB1, THBD, ABCG1, SELP, MDM2, CAT, MYC, CD163, F7, CTSD, OLR1, AR, SLC2A4, ALDH3A1, TUBB1, ABCC1, TOP2A, RASSF1, INSR, CASP3, SLC6A4, ESR1, ICAM1, RXRA, VCAM1, MTTP, MAPK8, MAPK1, HK2, IRF1, HIF1A, FOSL2, TP53, ADRA2A, CXCL8, CDKN1A, CDKN1A, CAT, CTSD
Lasso	ABCG1, CDKN1A, CXCL10, ESR2, F3, MDM2, MMP9, NR1I3, PCNA, SLC2A4, TUBB1, VCAM1

## Data Availability

The data used in this study are publicly available from several databases. Specifically, the Traditional Chinese Medicine Systems Pharmacology Database (TCMSP, available online: https://old.tcmsp-e.com/tcmsp.php (accessed on 29 January 2025)) provides comprehensive information on the pharmacological properties of Chinese medicinal herbs. Additionally, the BATMAN-TCM (available online: http://bionet.ncpsb.org/batman-tcm (accessed on 29 January 2025)) database offers tools for the analysis of bioactive compounds and their targets in traditional Chinese medicine. The SuperPred database (available online: http://superpred.net (accessed on 29 January 2025)) is utilized for predicting the biological activity of compounds. The high-throughput datasets generated and/or analyzed during the current study are available in the GEO database (available online: https://www.ncbi.nlm.nih.gov/geo/ (accessed on 29 January 2025)). All original data can be accessed freely online.

## Supplementary Materials

The following supporting information can be downloaded at: https://www.mdpi.com/article/10.3390/ph18020227/s1, Figure S1. Re-docking of the co-crystallized ligand: (a) CDKN1A (PDB ID: 1AXC)-Microstegiol. (b) NR1I3 (PDB ID:1XV9)-pentadecanoic acid. (b) TUBB1 (PDB: 1XVP)- pentadecanoic acid.
