# Supplementary material for: An Integration of RNA Sequencing and Network Pharmacology Approaches Predicts the Molecular Mechanisms of the Huo-Xue-Shen Formula in the Treatment of Liver Fibrosis
Source: Pharmaceuticals (Basel). 2025 Feb 7;18(2):227. doi: 10.3390/ph18020227 (PMC11859937; doi:10.3390/ph18020227)
Supplement: Supplementary file 1 [file pharmaceuticals-18-00227-s001.zip › pharmaceuticals-3389525-supplementary.pdf]

Supplementary Materials

Figure S1

a

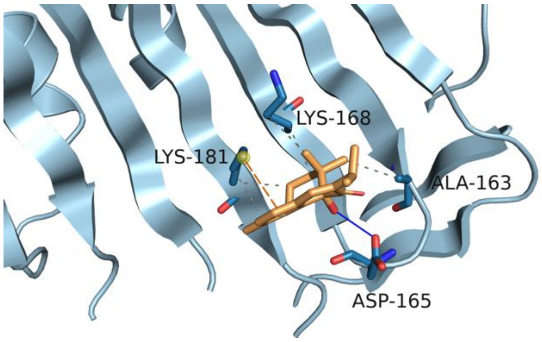

b

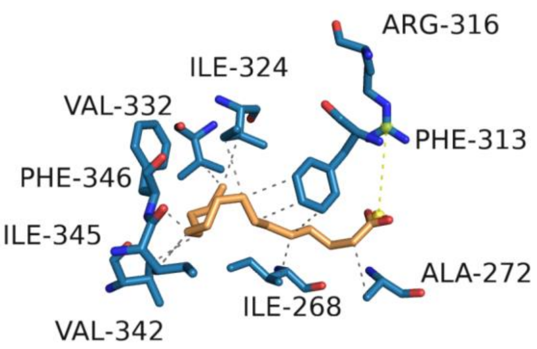

c

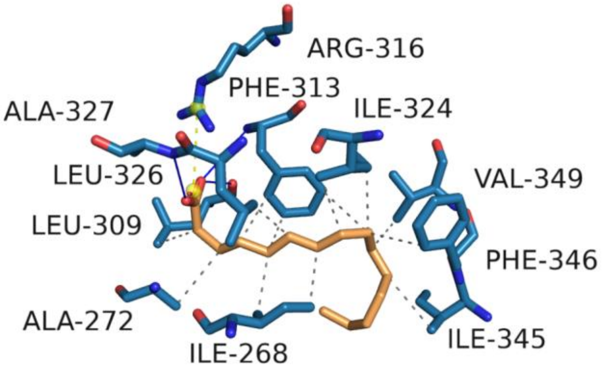

Figure S1. Re-docking of the co-crystallized ligand: (a) CDKN1A (PDB ID: 1AXC)-Microstegiol. (b) NR1I3 (PDB ID:1XV9)-pentadecanoic acid. (c) TUBB1 (PDB: 1XVP)- pentadecanoic acid.
